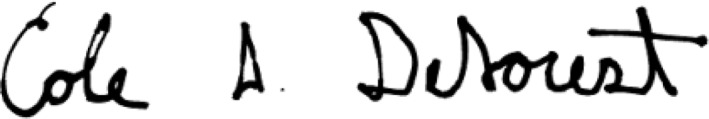# Introduction to Editorial Board Member: Professor Kristi S. Anseth

**DOI:** 10.1002/btm2.10117

**Published:** 2018-10-07

**Authors:** Cole A. DeForest

**Affiliations:** ^1^ University of Washington

1

In this issue of *Bioengineering and Translational Medicine*, we are pleased to introduce our Editorial Board Member, Prof. Kristi S. Anseth. Prof. Anseth is a Distinguished Professor and the Tony Tisone Endowed Chair in Chemical and Biological Engineering, Associate Professor of Surgery, and the Associate Director of the BioFrontiers Institute at the University of Colorado Boulder. She was the first engineer to be named a Howard Hughes Medical Institute (HHMI) Investigator and is one of a select few individuals elected to all three United States National Academies (Sciences, Engineering, and Medicine) as well as to the National Academy of Inventors for her major contributions to the fields of biomaterials, tissue engineering, and regenerative medicine.

Prof. Anseth earned her B.S. in Chemical Engineering with Highest Distinction from Purdue University where she performed undergraduate research in the laboratory of Prof. Nicholas Peppas. She completed her PhD in the Chemical Engineering department at the University of Colorado Boulder under the advisement of Prof. Christopher Bowman; her doctorate was completed in just 28 months and resulted in 10 first‐author publications. Upon graduation, she assumed postdoctoral research at the Massachusetts Institute of Technology with Prof. Robert Langer. In 1996, after just 1 year away from Boulder, she returned to her doctoral alma mater as faculty in the Department of Chemical Engineering at the University of Colorado.

**Figure 1:**
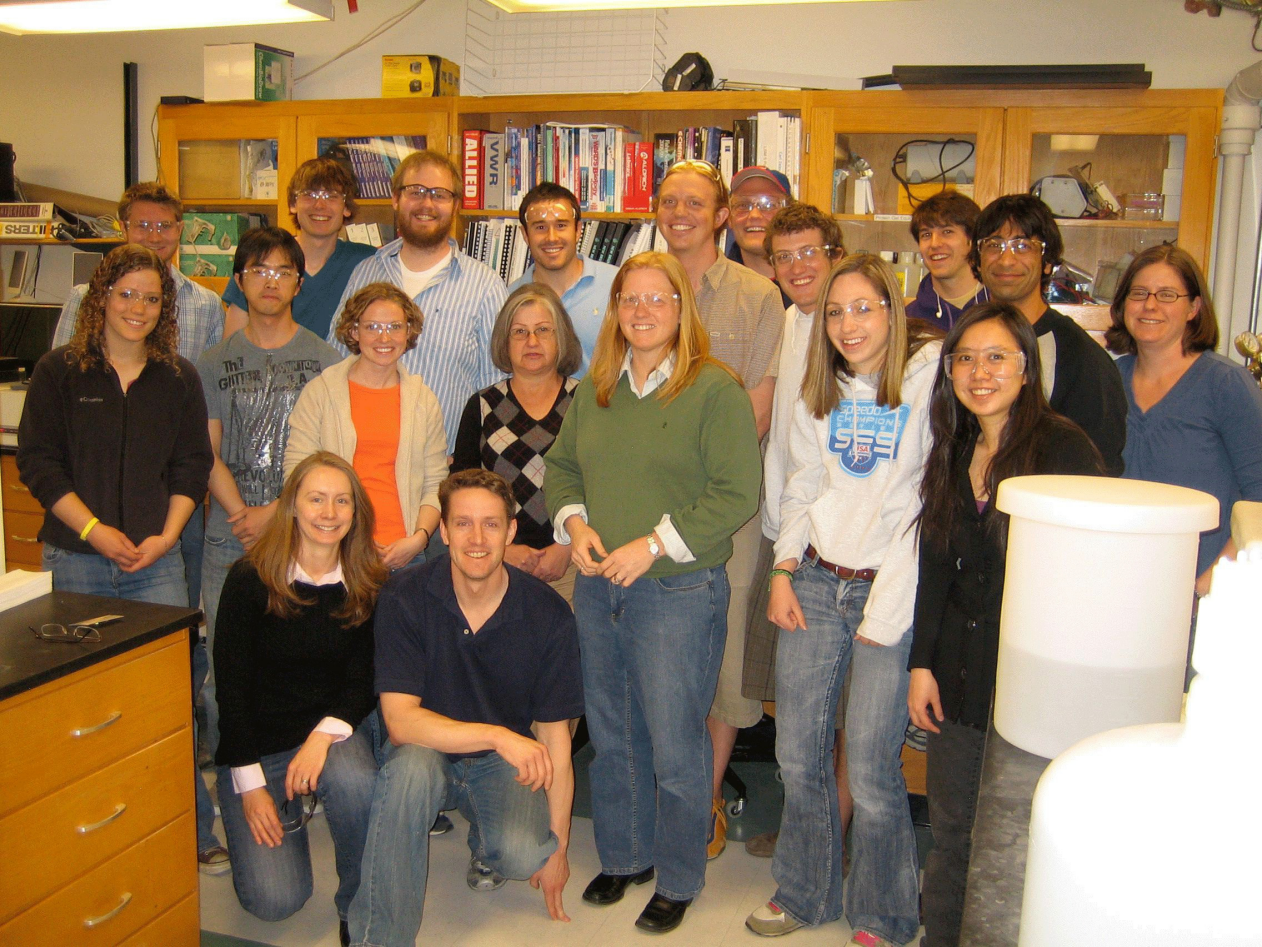
Prof. Kristi Anseth (front row, center) at an impromptu lab gathering in celebration of her renewed appointment as an HHMI Investigator in 2010

Repurposing traditional polymer chemistry approaches for applications in the biological sciences, a theme that permeates much of her research, Prof. Anseth's early efforts sought to synthesize and characterize biodegradable polymeric materials for controlled drug release. Pairing reaction kinetics and polymer network theory, she established powerful statistical models to predict bulk material degradation over time.^1^ These meticulously derived models are still used today and helped earn Prof. Anseth early‐career awards from the Camille and Henry Dreyfus Foundation, the David and Lucile Packard Foundation, the National Science Foundation (NSF), and the National Institutes of Health.

Prof. Anseth has demonstrated that many of the same polymer chemistry methodologies used for controlled drug delivery can also be exploited to synthesize scaffolds for three‐dimensional (3D) cell culture. She was among the first to show that traditional photopolymerization reactions could be performed in the presence of live cells,^2^ and that cells maintained in 3D behave more like they do in vivo than in two‐dimensional culture.^3^ By functionalizing otherwise bioinert polymeric scaffolds with bioactive small molecules, peptides, and proteins, she has facilitated evolution beyond biomaterials that passively support cell viability to those that actively promote specific functions.^4^, ^5^ These efforts helped earn Prof. Anseth recognition as an HHMI Investigator, an Alan T. Waterman Award from the NSF, and the Allan P. Colburn Award from the American Institute of Chemical Engineers (AIChE).

**Figure 2:**
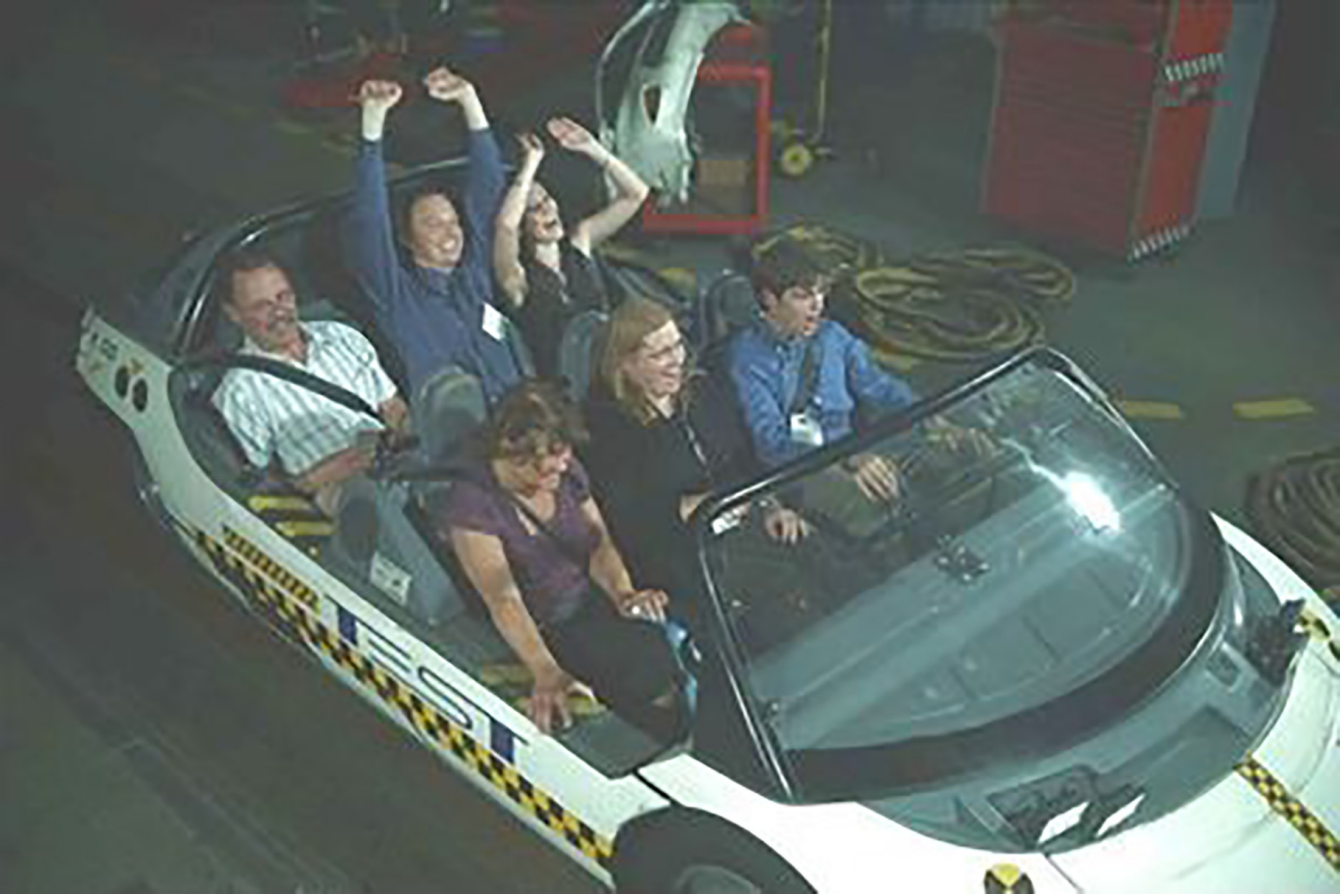
Prof. Anseth (front row, center) takes advantage of the Society for Biomaterials' 2011 Annual Bash to get behind the wheel with trainees on Walt Disney World's Test Track ride

Recognizing that biological tissue composition varies tremendously over time, particularly during development and disease, Prof. Anseth has pioneered the creation of dynamic materials whose physical and chemical properties can be externally modulated on demand and in the presence of living cells.^6^, ^7^, ^8^ Utilizing light to alter materials with control in both time and 3D space, Prof. Anseth has developed a suite of advanced materials that enable unique experiments in “4D biology.”^9^ These efforts have earned Prof. Anseth recognition as one of *Popular Science*'s “Brilliant 10” and AIChE's “One Hundred Chemical Engineers of the Modern Era.”

Prof. Anseth's innovative approach to engineering biomaterials continues to yield new understandings in fundamental biological processes as well as translational advances in a variety of applications. Taking advantage of photodegradable materials that can soften with the flick of a switch, she was the first to demonstrate the concept of “mechanical memory” whereby future cellular decisions are influenced heavily by past culture conditions.^10^ Her materials‐based approaches have also led to new solutions for engineered joint repair, diabetes treatment, and heart valve replacement. These advances have resulted in named lectureships at Princeton, Rice, Johns Hopkins, and more than 30 other universities.

Throughout all of her research success, Prof. Anseth makes a special point to give back to the community. She served as the President of the Materials Research Society, the Chair of the Frontiers of Engineering Program for the National Academy of Engineering, and was recently elected as a member of AIChE's Board of Directors. She has chaired and organized many conferences and symposia to promote graduate education and science alike and has helped identify future opportunities in biomaterials research on NSF steering committees. In addition to *Bioengineering and Translational Medicine*, Prof. Anseth served on the editorial boards for journals including *PNAS*, *Biomacromolecules*, and the *Annual Review of Chemical and Biomolecular Engineering*.

Above all, Prof. Anseth is a fantastic educator. She has mentored over 45 graduate students and 25 postdoctoral fellows in her laboratory, placing an even split of her trainees in academic and industry positions. Her uncanny ability to successfully educate both in the lab and the classroom has been recognized through the highest teaching awards given by her department, college, and university, as well as through a Camille Dreyfus Teacher‐Scholar Award. She remains a life‐long mentor to each of her mentees, always willing to take a call to celebrate the highs or help move past the lows. On behalf of her past, present, and future trainees, I express my sincerest gratitude for Kristi's scientific excellence and her unmatched selflessness to the biomaterials community.